# Can we understand and improve poorer cancer survival in rural-dwellers?

**DOI:** 10.3399/bjgpopen19X101646

**Published:** 2019-05-15

**Authors:** Peter Murchie, Rosalind Adam, Rose Wood, Shona Fielding

**Affiliations:** 1 Professor of Primary Care, Institute of Applied Health Sciences, University of Aberdeen, Aberdeen, UK; 2 Senior Clinical Fellow, Institute of Applied Health Sciences, University of Aberdeen, Aberdeen, UK; 3 Clinical Academic Fellow, Institute of Applied Health Sciences, University of Aberdeen, Aberdeen, UK; 4 Senior Lecturer in Medical Statistics, Institute of Applied Health Sciences, University of Aberdeen, Aberdeen, UK

**Keywords:** Cancer, Geographic Factors, Rurality, Mortality, Socioeconomic Factors, General Practice

## Introduction

In this article, the concept of geographical cancer equality is described and strong evidence that rural populations have poorer cancer outcomes is highlighted. Currently, hard evidence is lacking for what causes poorer outcomes in rural populations with cancer, and this hinders efforts to address this striking health inequality. Current trends in ‘big data’ science and systems to assess the quality of universities’ research output could discourage the very kind of research that will truly discover why rural communities fare worse with cancer. Smaller local studies, employing mixed research methods and using technology in innovative ways, offer the way forward and will signpost policies and interventions with real potential to redress urban–rural cancer inequality.

## Can we understand and improve poorer cancer survival in rural-dwellers?

Rural-dwellers have poorer cancer survival than those living in cities. In a recent systematic review, we identified 39 studies from seven countries, the majority reporting poorer survival in rural areas.^1^ Place of residence is a major source of inequity worldwide, but compared to other sociodemographic factors affecting cancer outcomes, geographical cancer inequalities are under-researched and barely understood. One problem is that current insights into geographical inequity derive from population-based studies and big data research. It is accepted practice for data scientists to offer explanations for significant associations in their data. In the case of rural residence and increased cancer mortality, the usual explanation is almost too obvious: rural-dwellers have poorer access to health services. In reality, geographical inequality is likely multi-factorial, driven by patient, topographical, and cultural factors, as well as just service organisation. Potentially modifiable local factors are obscured in big data. Here, we argue that collected insights from robust local and primary care-based studies offer the best chance to explain and tackle rural cancer inequality, with opportunities to extrapolate to other complex health problems impacted by geography.

## Big data, big assumptions, small insights

The impact of geography on cancer survival has been researched worldwide. First, in 1990, a study based on 2589 breast cancer cases notified to the South Australian Central Cancer Registry between 1980–1986 found poorer survival in non-metropolitan areas.^2^ In 2000, an analysis of 63 976 Scottish people diagnosed with one of six common cancers between 1991–1995^3^ found increasing distance from a cancer centre was associated with less chance of diagnosis before death and poorer survival from cancer. More recently, an even larger English study linking several national datasets studied 737 495 people diagnosed with one of eight cancers between 2006–2010, and reported those living >30 minutes from their GP were at greater risk of having cancer diagnosed as an emergency, were less likely to be diagnosed before death, and were less likely to have screen-detected cancer.^4^ All three studies explained their results in terms of poorer access to services and a tendency for rural-dwellers to take longer to present to their GP.

Notably, however, the much more comprehensive, but regionally-based and primary care-led, Northeast and Aberdeen Scottish Cancer and Residence (NASCAR) study linked extensive data from multiple sources for 12 339 people diagnosed with one of eight cancers between 2007–2013.^5^ NASCAR found those most remote from cancer centres were actually referred from primary care most quickly and did not have more advanced disease at presentation. The most remote patients were also diagnosed and treated most quickly, receiving the same treatment as those living closer-by. This did not, however, improve outcomes, as the most remote patients continued to have the poorest 1-year survival. These findings effectively refute the commonest explanations for the rural cancer outcome disadvantage, for Northeast Scotland at least, and raise an important question: if access to cancer diagnosis and treatment is no worse for rural patients, why is mortality higher in this group? Consequently, healthcare professionals and policymakers are faced by a bewildering array of possible targets on which they might intervene to improve cancer survival in rural dwellers.

There are many potential advantages of conducting research using geospatial and routinely collected ‘big’ healthcare data. However, as data-linkage potential grows, and more elaborate and technologically-driven research methods become possible, there is a danger that studies of rural–urban cancer disparity could also become larger, more comprehensive, and more complex without necessarily becoming more illuminating. In the UK, a move towards research using big data has been coupled to targets within the Research Excellence Framework, which drives researchers to produce work that is world-leading in terms of originality, significance, and rigour. Producing research of international significance is an important aspiration for academic institutions, but risks discouraging rigorous and illuminating research at local and regional levels. Studies, such as those examining the organisation of regional cancer services and the link with cancer outcomes, become unattractive for fear of having insufficient scale or impact. This is a particular concern for rural cancer inequality.

## Ways forward

Rural cancer inequality is observed throughout the world, but is also a complex local issue. True understanding will require rigorous and innovative local studies which can be compared and contrasted. The strength of local data resides in its granularity. Combining, for example, routine health service data with detailed narrative accounts from primary care-held medical records enables researchers to reach beyond rudimentary statistical associations, and achieve clearer insights into true mechanisms in real-world health services.^6^ Emerging research methods from data science, such as natural language processing, may afford ever greater insights in future when applied to local data, with potential to do simultaneous research in different parts of the world.

The specific topography of different UK regions, and corresponding area-level organisation of healthcare services, directly influences the experience of cancer patients.^7,8^ International comparisons are difficult; rurality in Australia or Canada, for example, is on a different scale to that of western Europe, and is further complicated by cultural factors in native populations.^9^ However, methods to account for this are now emerging.^10^ Although unresearched, the rural cancer disadvantage in developing countries is likely to be even larger, due to poor infrastructure and complications from communicable diseases. Going forward, improved classification systems and the application of appropriate data-science to compare, rather than combine, data offer potential.

Geographic information computer systems capture and compare data on the precise global position of cancer patients and their health services, and could lead to exciting ways to compare health service structure and outcomes in different parts of a country, or between countries. Appropriate technological innovation may also enable translation of knowledge from developed to developing countries. Effective interventions to promote geographical cancer equality will also likely utilise digital technologies, and some evidence already exists that rural health care delivery can be improved by technology.^11^

More fundamentally, the question remains about what observed cancer inequalities actually mean. It could be that rural populations are disadvantaged by easily remediable health service delivery factors which can be discerned and overcome with high quality data and realistic healthcare interventions. Alternatively, geographical cancer equality could be impossible to achieve. More controversially, observed and apparent inequalities could actually reflect superior care; rural GPs, for example, are more likely to excise melanomas with no evidence of compromised outcomes.^7^ Poorer 1-year cancer survival rates could actually reflect more realistic attitudes to admissions and further treatment.^12^ Recognising how a patient’s geographical location influences their healthcare choices is central to personalised and realistic medicine, which will shape future health policy and care delivery.

To tackle geographical cancer inequality, we must reach beyond simply exploring statistical associations to a much richer context-specific research combining quantitative and qualitative methods. Some important qualitative studies, in several countries, have been conducted which were not included in our meta-analysis and narrative synthesis.^1^ Vital, too, is meaningful engagement with people in rural communities to truly understand their priorities for research and change.

Physical remoteness conferred by rurality is unalterable. It is vital for governments, researchers, and industry to work with rural-dwellers to design future rural cancer services. Finally, rurality or rural residence is more than a physical reality, and anthropological research is required to understand how the cultural attributes and attitudes of rural populations influence cancer outcomes.
